# The first case report of Haim Munk disease with neurological manifestations and literature review

**DOI:** 10.1002/ccr3.4802

**Published:** 2021-09-07

**Authors:** Mehdi Moghaddasi, Mohammadreza Ghassemi, Mohammad Shekari Yazdi, Seyed Amir Hasan Habibi, Nafiseh Mohebi, Azadeh Goodarzi

**Affiliations:** ^1^ Department of Neurology Rasool Akram Medical Complex Iran University of Medial Sciences (IUMS) Tehran Iran; ^2^ Department of Dermatology Rasool Akram Medical Complex Iran University of Medical Sciences (IUMS) Tehran Iran

**Keywords:** demyelinating disease, Haim‐Munk syndrome, palmoplantar keratoderma, Papillon‐Lefevre syndrome, periodontitis

## Abstract

HMS can have neurologic MS like manifestations. It is urgent to do more research and report probable unknown associations of HMS for its better management.

## Graphical abstract

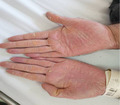

## 1 INTRODUCTION

Papillon‐Lefevre syndrome and its phenotypic variant, Haim‐Munk syndrome, are syndromic palmaplantarkeratodermas. This is the first report of neurologic manifestations of MS in a known case of HMS. The patient was treated with methyl prednisolone pulse therapy with a cumulative dose of 5 grams.

Papillon‐Lefevre syndrome (PLS) is an autosomal recessive syndromic generalized palmoplantar keratoderma (PPK). It was first described by Papillon MM and Lefevre P in 1924.^1^ Its prevalence is 1–4 per million and has no gender predilection.^2^, ^3^ Clinically it has a transgradient PPK. PLS patients have a loss of function mutation in cathepsin C gene (CTSC). Cathepsins could be Cysteine, Serine or Aspartic. Cathepsin C belongs to Cysteine type cathepsins.^4^ CTSC mutation leads to periodontitis and patients lose their teeth during childhood and later lose their permanent teeth.^5^ They can have pseudoainhum and psoriasiform plaques and are predisposed to pyogenic infections. Haim‐Munk syndrome (HMS), first described by Haim and Munk in 1965,^6^ is the phenotypic variant of PLS with the CTSC gene mutation.^7^ Haim‐Munk characteristic signs are onychogryphosis, arachnodactyly, acro‐osteolysis, flat foot and permanent flexion contractures.^8^

Periodontitis in Papillon‐Lefévre syndrome and Haim‐Munk syndrome arises from a failure to eliminate periodontal pathogens because of cathepsin C deficiency. Recruitment and accumulation of hyperactive/reactive neutrophils and a reduced antimicrobial capacity lead to a locally destructive chronic inflammatory cycle in Papillon‐Lefévre syndrome and Haim‐Munk syndrome.^9^ There has been some improvement in impaired autophagy, caused by insufficient lysosomal function, after introducing cathepsin C in fibroblast cell culture.^10^ Haim‐Munk syndrome (HMS) could also be erythrodermic or be accompanied with destructive arthritis.^11^, ^12^ Retinoids such as acitretin, topical keratolytics, antibiotics for oral and pyogenic infections and dental care can be used as treatment for PLS and HMS.^13^ A tabular literature review of PLS treatment can be found in Tambe L, Dixit M, Patil N. literature review.^14^

### 1.1 Neurologic manifestations

Mental retardation and psychotic depression have been reported in PLS.^15^ Dural and choroid plexus calcifications are also found in patients with PLS.^8^ There is a report of convulsion due to multiple cerebral abscesses.^16^ Finally, there have been reports of abdominal epilepsy in a patient with PLS.^17^

We were unable to find any neurological manifestations of Haim‐Munk syndrome in peer‐reviewed literature.

## 2 CASE REPORT

The patient is a 42‐year‐old married Muslim man from Ardakan, a province of the city Yazd, in Iran. He has one healthy daughter and comes from a middle‐class background. From early infancy, when he was 6 months old, he has been suffering from transgradient PPK. He lost his deciduous and later his permanent teeth. At the age of 15, he lost all of his teeth, and hence became edentulous. At the same time, he suffered PPK with the hyperkeratosis of elbows and knees. His pathology report from 2009 shows hyperkeratosis, acanthosis and perivascular lymphohistiocytic infiltrate in upper and mid dermis, compatible with PLS hyperkeratotic skin lesions.

He is a first‐born child and has two siblings; one sister and one brother. His siblings are healthy and have no sign of PPK. His parents were first‐degree cousins. His father died from brain tumor and his mother suffers from seizures and is on anticonvulsive and antidepressant medications. Neither his parents nor his siblings suffered from PPK.

At the age of 15 the patient began receiving corticosteroids and Neotigason. Due to the development of arthralgia and bone pain, his physician decreased the dose of Neotigason. For the past years, he has been on Roaccutane 20 mg fort wo to three times a week and has had stable skin lesions.

In dermatologic examination, the patient had transgradient PPK with knee and elbow involvement. He also suffered from flexion contracture of toes, onychogryphosis, and arachnodactyly, all of which are characteristics of Haim‐Munk syndrome.

### 2.1 Neurologic manifestations

His present illness began in January 2020 when he developed fever, malaise and sleepiness. Additionally, he developed right‐sided weakness that improved spontaneously after a week. The exact same episode repeated itself 8 months later in mid‐September of 2020. Again, his condition improved spontaneously. Furthermore, the patient complained from tiredness after walking in the prior2‐3 months.

After the last episode in September 2020, the patient was admitted to the neurology ward of Rasul‐e‐Akram hospital in Tehran, Iran. In his neurologic examination, he had spastic gait with a wide base, increased deep tendon reflexes (DTR), upward plantar reflexes and bilateral dysmetric finger to nose tests. Ophthalmologic exam was normal.

Laboratory tests, including acute phase reactants and viral markers, were unremarkable. Serum NMO and MoG Ab were negative. CSF fluid analysis was normal as well. A positive oligoclonal band test was performed with a delay, which confirmed the diagnosis.

Brain MRI showed multiple bilateral non‐enhancing hypersignal lesions mostly in periventricular, subcortical, juxtacortical, infratentorial (pons and cerebellar peduncles) regions in T2 and FLAIR sequences. In cervical MRI, C1‐C2 and C2‐C4 levels had multiple T2 and STIR hyperintense patchy foci without enhancement.

According to the MRI findings, with an impression of demyelinating disease, he received methyl prednisolone pulse therapy with a cumulative dose of 5 gr.

Additionally, he suffered from depression and irritability and hurt himself by banging his head when he was angry.

**FIGURE 1:**
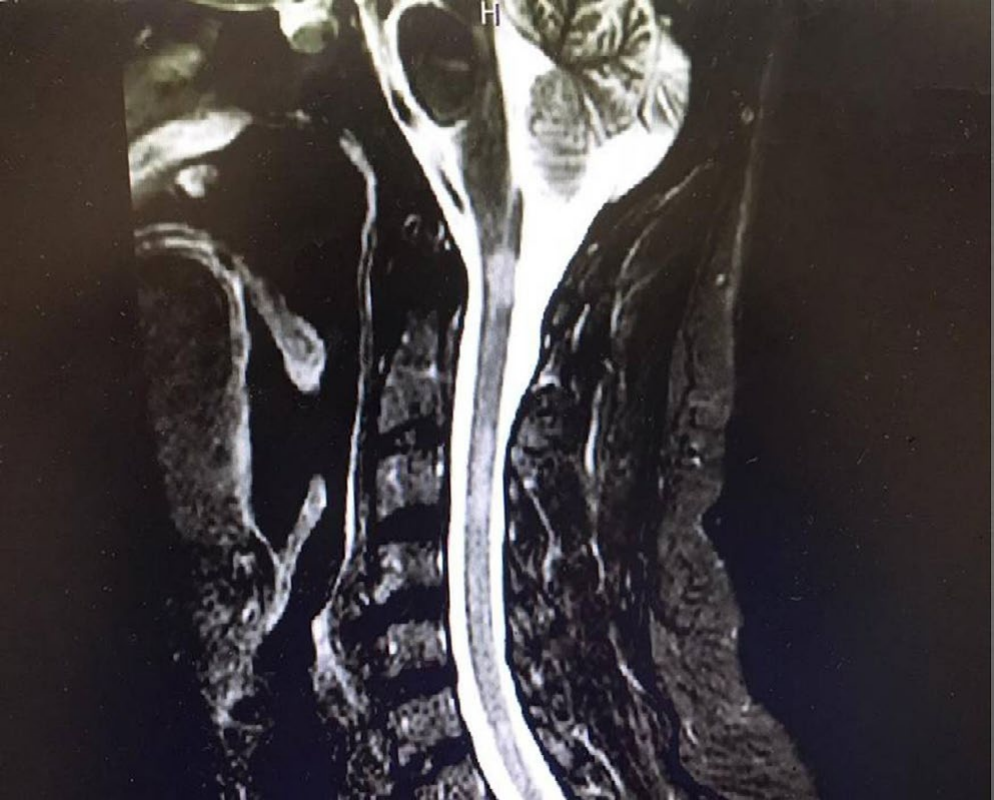
T2‐STIR cervical MRI shows multiple bright lesions compatible with demyelinating disease (multiple sclerosis)

**FIGURE 2:**
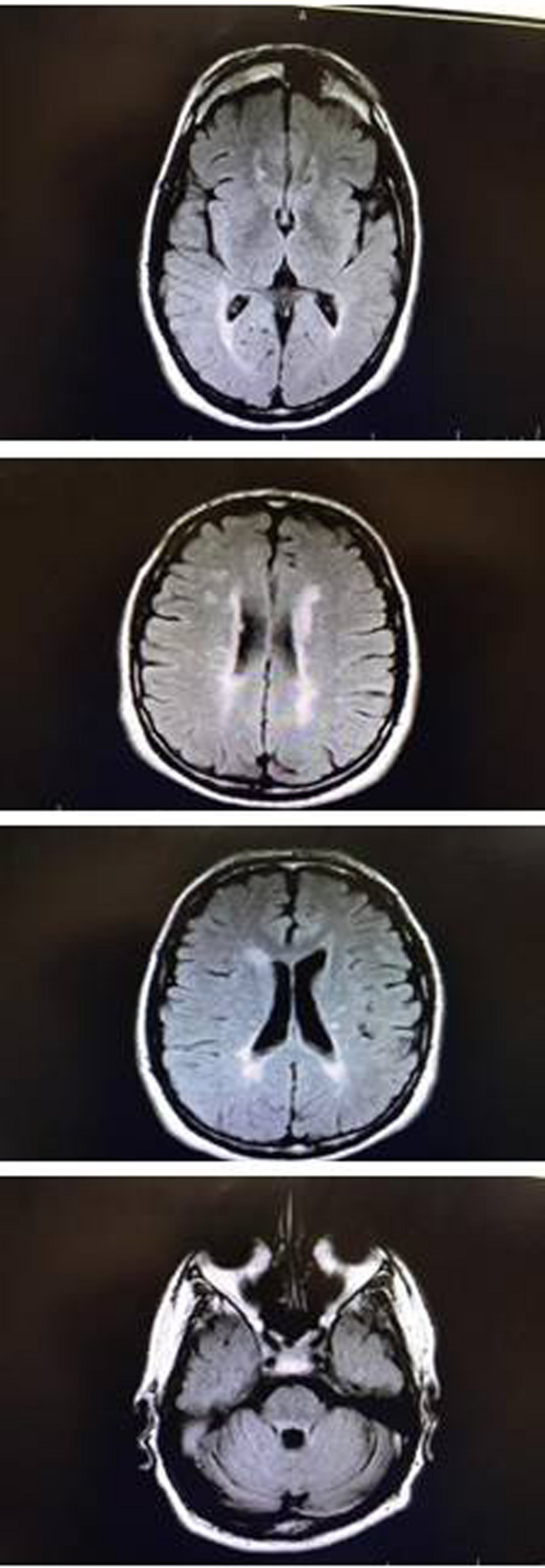
Flair T2 sequences of brain shows multiple bright lesions compatible with demyelinating disease (multiple sclerosis)

**FIGURE 3:**
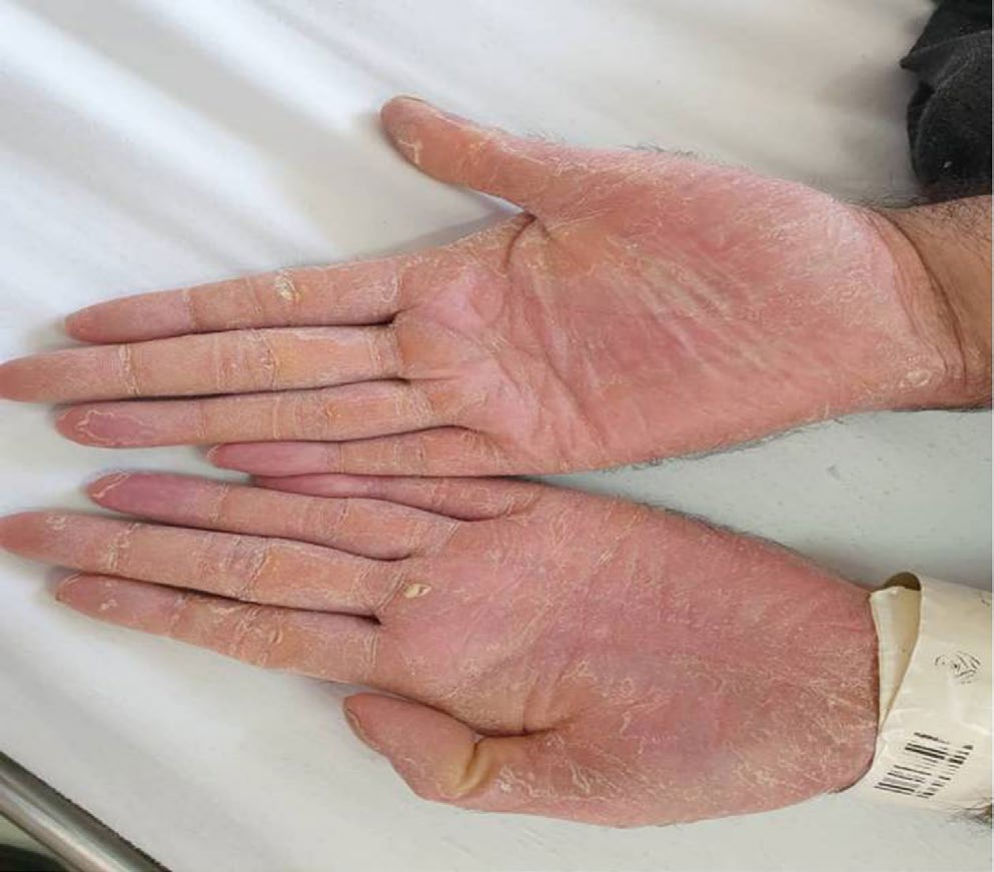
Arachnodactyly

**FIGURE 4:**
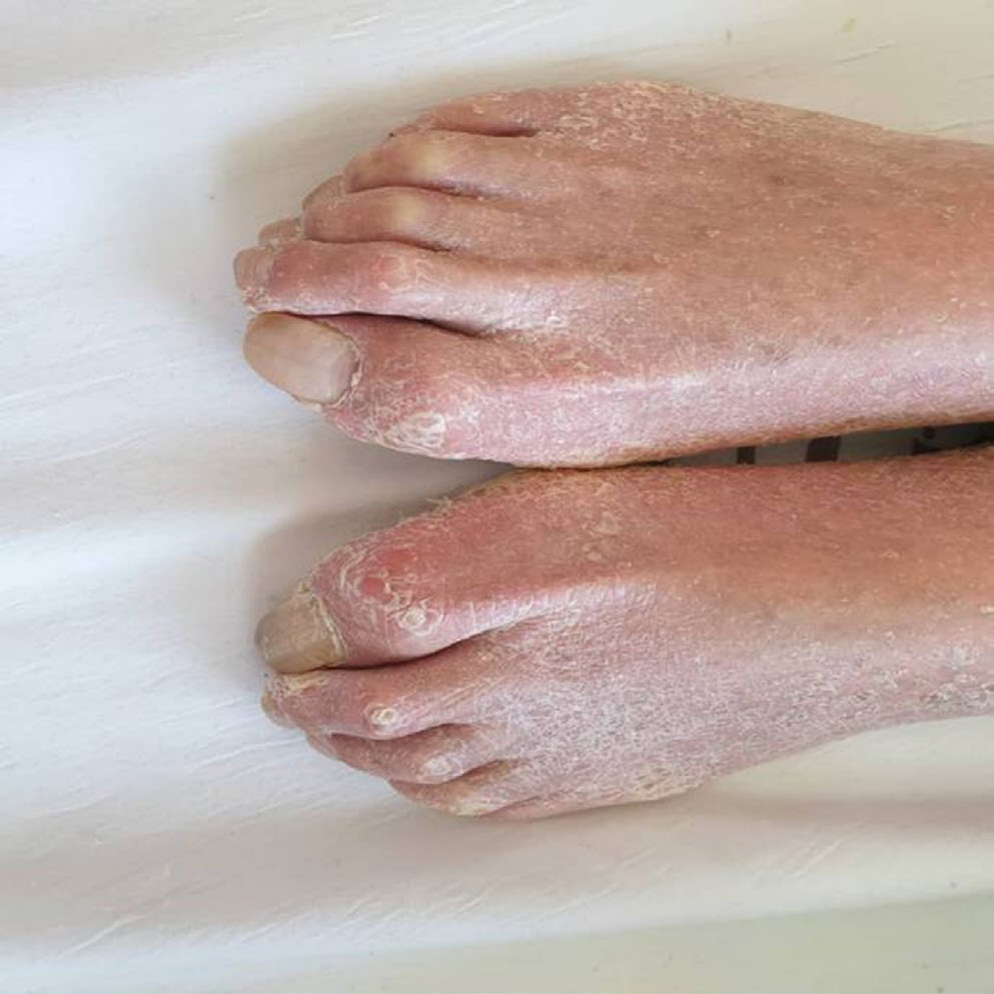
Flexion contracture of the toes

**FIGURE 5:**
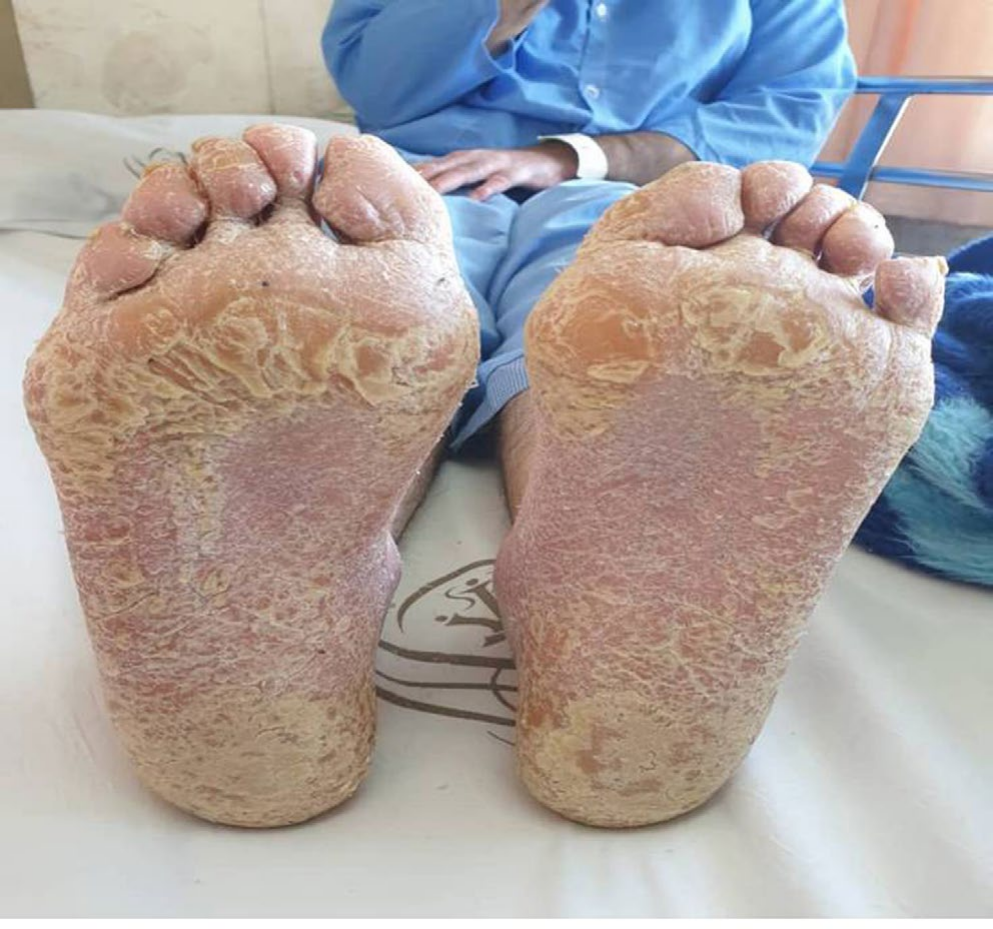
Plantar hyperkeratosis

**FIGURE 6:**
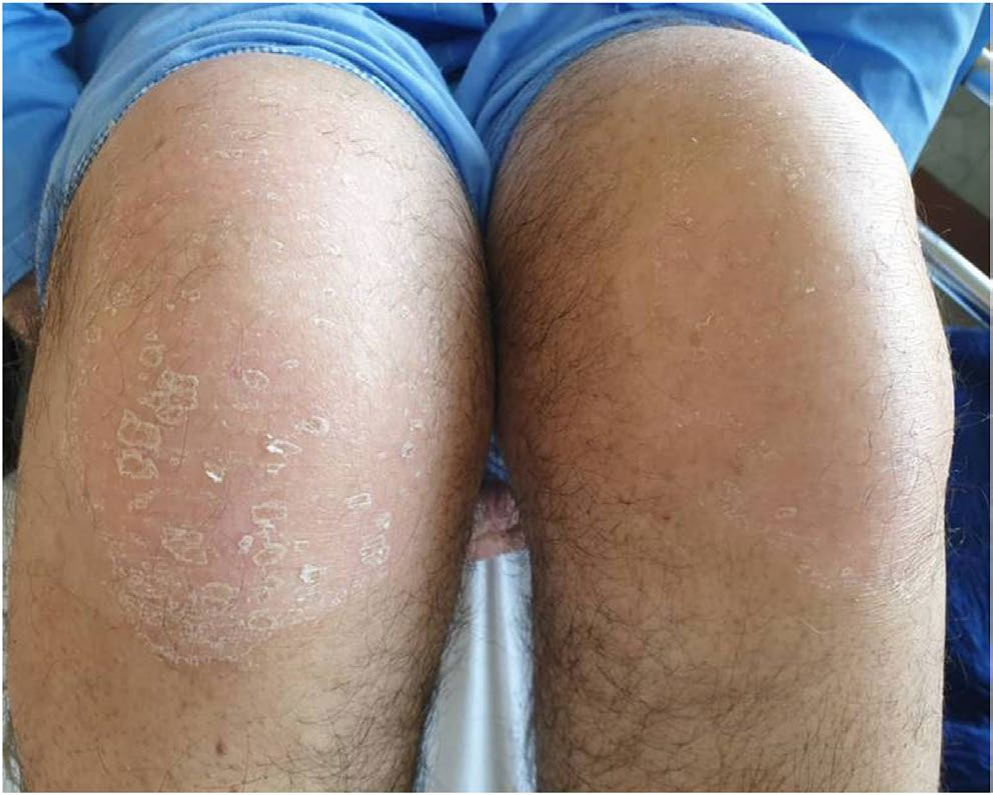
Knee hyperkeratosis

After discharge the patient has been put on pantoprazole 40 mg QD, Vit B1 300 mg QD, Tab Ca‐D QD, Pearl Vit D3 50000 U weekly, Tab Hydroxychloroquine 200 mg QD, Tab Roaccutane20 mg twice weekly and Eucerin + urea 5% cream. Figures 1, 2, 3, 4, 5, 6 show neurological and dermatological manifestation of the reported case.

## 3 DISCUSSION

In addition to periodontitis and PPK, HMS has arachnodactyly, acro‐osteolysis, atrophic changes of the nails (and in some sources onychogryphosis), and a radiographic deformity of the fingers. Additionally, some regard flat foot (Pes Planus) as part of the criteria for HMS.^18^, ^19^, ^20^

The first cases of HMS originated from Cochin, India. Hypotheses for HMS include parental consanguinity and a complex autosomal recessive inheritance.^21^

There have been many reports of PLS from Iran.^22^, ^23^, ^24^, ^25^, ^26^, ^27^ Google scholar and pub med searches for the words “Haim‐Munk” and “Iran” did not yield any relevant results.

Our patient had typical features of PLS, i.e. being edentulous and having transgradient PPK. Furthermore, he displayed additional features of HMS, i.e. arachnodactyly, onychogryphosis, and flexion contracture of toes and fingers. The psoriasiform lesions should be differentiated from typical psoriasis, especially in regards to diagnostic and therapeutic approach.^28^, ^29^, ^30^, ^31^, ^32^, ^33^

Our patient had two episodes of fever, malaise and sleepiness that healed spontaneously and has had tiredness for the last months. MRI showed multiple bilateral hypersignal T2/FLAIR lesions in periventricular, subcortical, juxtacortical, infratentorial plus multiple patchy hypersignalT2/STIR lesions in C1‐2 and C2‐4 levels with a final diagnosis of demyelinating disease (Multiple sclerosis). He also suffered from depression and irritability.

Cathepsin C deficiency, which is prevalent in PLS and HMS, has not shown to affect immune dysregulation and autoimmunity. However, other cathepsin deficiencies, such as cathepsin D, is shown to cause neuropathogenesis, such as seizures and demyelination of white matter.^4^

In our opinion, our patient could have mutations in both cathepsin C and other cathepsins, like cathepsin D, or a novel cathepsin C mutation, resulting inneurologic manifestations. This requires further investigations.

Finally, we reviewed and compared latest pubmed publications on PLS and HMS in the past10 years. We summarized the most important and relevant articles on phenotypic variant, Haim‐Munk syndrome, in Table 1.

**TABLE 1:** Recent reports of PLS/ HMS and their clinical characteristics

source	periodontitis	PPK	Hyperkeratosis	Infection	Other
Adamski Z., et al. 2020^34^	+	+		Purulent URI & Appendicitis	Ear lobe hypoplasia
Alsaif FM. et al. 2019^35^	+	+			Nodular BCC
AbouChedid JC. et al. 2019^36^	+	+	Dorsum of hands & feet		
Yousry YM. et al. 2018^37^	+	+			
Silva TS. et al. 2018^38^	+	+		Pneumonia, Gasteroenteritis	
Fageeh HN 2018^39^	+	+			
Lingeswaren A., Gopal SD. 2018^15^	+	+	psudoeinhum		Psychotic depression
AlBarrak Z M., et al. 2016 (5 cases)^40^	+ (5)	+ (4)	Knees (4), Toes (3), dorsal fingers (2)		
Iqtadar S, et al., 2015^41^	+	+		Pyogenic liver abscess, Gastroenteritis, UTI, Respiratory infection	Pyrexia of unknown origin, Anorexia,
Bhavsar M V., et al., 2013, (2 siblings)^42^	+	+	Dorsal of hands & feet, elbows, knees		Nail dystrophy, transverse nail groove, pointed and clawed fingers in one case
Mercy p., et al. 2013^43^	+	+	Elbow, knee	Recurrent skin infections, liver abscess	
Sharma A., et al., 2013^44^	+	+	Dorsum of hand joints		
Valeshabad AK., et al. 2012, (6 cases)^25^	+ (6)	+ (6)	Ext. malleolus (5), Knee & elbow (4), dorsal fingers (3), thigh (2)		Depression (6), Mental retardation (4)
Khan FY., et al. 2012, (2 cases)^45^	+	+	Dorsum of hand and feet and discolored nail in one of them		
Sachdeva S., et al., 2012^46^	+	+	Dorsum of hands and feet		
Muppa R., et al. 2011^47^	+	+	Dorsum of hands and feet		
Veerabahu BG., et al. 2011^48^	+	+	Dorsum of hands and feet, knee, elbow		Malnourish, anemic
Pahwa p., et al. 2010^19^	+	+	Elbow, lat. Malleolus, Achilles tendon	Recurrent skin infections	Pes planus, nail transversegroove and pitting
Yasar, Halit et al., 2015^17^	+	+			Nausea, epigastric pain
Kanthimathinathan HK., et al., 2013^16^	+	+		Brain abscess	

As is evident from Table 1, there have been reports of psychiatric manifestations like depression and some cases of mental retardation. However, this is a case of PLS/HMS that has MS like signs and symptoms.

## 4 CONCLUSION

PLS/HMS can have different psychiatric and neurologic manifestations. They include depression, mental retardation, seizure and abdominal epilepsy. We report PLS/HMS with a demyelinating disorder (MS).

## CONFLICT OF INTEREST

The authors certify that there is no conflict of interest with any financial organization regarding the material discussed in the manuscript.

## AUTHOR CONTRIBUTIONS

MM and MG involved in conception and design of the work; MSY, SHH, NM, and AG involved in manuscript preparation and drafting the manuscript; MSY, SHH, NM, and AG involved in searching the literature; All authors involved in critical revision of the manuscript for content. All authors read and approved the final manuscript.

## CONSENT

Published with written consent of the patient.

## Data Availability

Data are available on reasonable request from the corresponding author.
